# Targeting CD162 for Life‐Threatening Cutaneous Chronic Graft‐Versus‐Host Disease: A Case Report

**DOI:** 10.1002/jha2.70342

**Published:** 2026-07-03

**Authors:** Alicia Lieberman, Ross McCauley, Maureen Ross, Philip McCarthy, Iming Cho, Simona Reed, Shih‐Yao Lin, Judy Chou, Shernan G. Holtan

**Affiliations:** ^1^ Roswell Park Comprehensive Cancer Center Transplant and Cell Therapy Program Buffalo New York USA; ^2^ Altrubio, Inc San Francisco California USA

**Keywords:** case report, CD162‐targeted antibody, chronic graft‐versus‐host disease, PSGL‐1, skin GVHD

## Abstract

Steroid‐refractory chronic cutaneous graft‐versus‐host disease (GVHD) causes substantial morbidity, prolonged hospitalization, and non‐relapse mortality after allogeneic hematopoietic cell transplantation. We report a young adult with multisystem chronic GVHD and recurrent life‐threatening inflammatory skin flares refractory to multiple standard therapies, with cumulative inpatient care exceeding 1 year. Under an institutional review board‐approved compassionate‐use protocol, he received a non‐cytotoxic monoclonal antibody targeting CD162 (P‐selectin glycoprotein ligand‐1). Treatment was associated with durable resolution of active cutaneous GVHD, corticosteroid reduction, improved mobility, and meaningful recovery in daily function and quality of life.

**Trial Registration:**

The author has confirmed clinical trial registration is not needed for this submission.

## 1 Introduction

Chronic graft‐versus‐host disease (GVHD) remains a leading cause of late morbidity and non‐relapse mortality after allogeneic hematopoietic cell transplantation [1, 2]. Skin is the most commonly affected organ and can become the dominant driver of suffering when disease is severe and inflammatory, causing pain, skin barrier disruption, infection risk, immobility, and prolonged exposure to systemic immunosuppression [3]. In the steroid‐refractory setting, treatment is especially difficult because patients often cycle through sequential therapies with incomplete responses, treatment‐limiting infections, and cumulative complications that are challenging to manage [4].

CD162 (P‐selectin glycoprotein ligand‐1, PSGL‐1) regulates leukocyte trafficking and T‐cell activation and is a biologically plausible target in GVHD [5, 6]. ALTB‐168 (aka neihulizumab) is an intravenous noncytotoxic monoclonal antibody directed against CD162 that selectively targets activated T cells studied in acute GVHD [7, 8]. ALTB‐268 is a second‐generation, higher‐potency antibody for the same target, formulated for subcutaneous (SQ) administration, and currently under investigation for refractory ulcerative colitis (NCT06109441). We report a young adult with severe multisystem chronic GVHD, dominated by life‐threatening refractory cutaneous disease, who achieved durable remission after compassionate‐use ALTB‐168 followed by ALTB‐268.

## 2 Case Presentation

**FIGURE 1:**
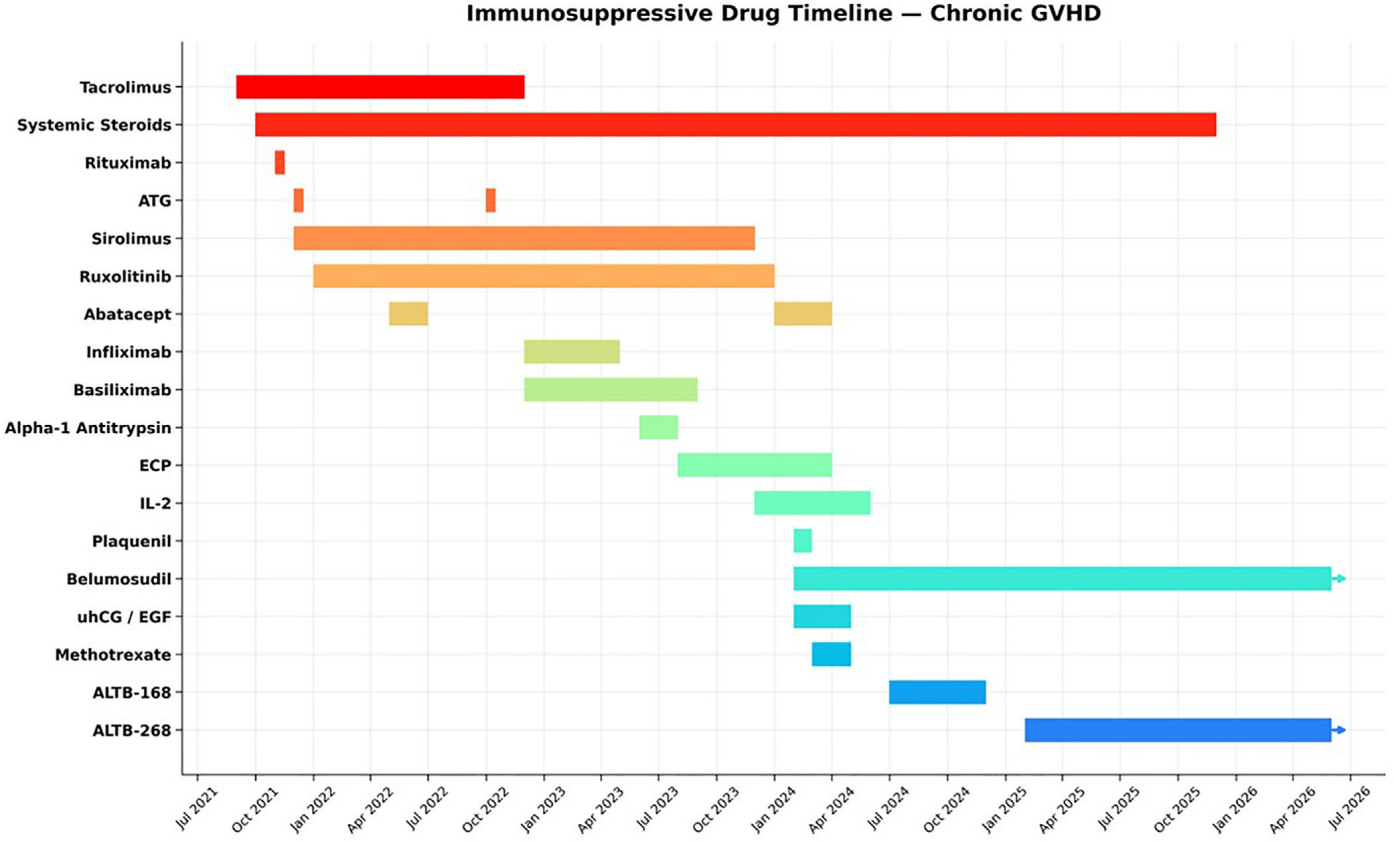
Immunosuppressive drug timeline.

A 19‐year‐old adult man with Philadelphia chromosome‐negative B‐cell acute lymphoblastic leukemia underwent HLA‐B‐mismatched donor peripheral blood stem cell transplantation in 2021 following conditioning with fludarabine, melphalan, and total body irradiation. GVHD prophylaxis was with tacrolimus, mycophenolate mofetil, and mini‐methotrexate. His early posttransplant course was complicated by biopsy‐proven acute GVHD involving skin, liver, and gastrointestinal tract treated with corticosteroids, anti‐thymocyte globulin, tocilizumab, alpha‐1 antitrypsin, lithium, infliximab, and basiliximab. His acute GVHD evolved into chronic multisystem GVHD with involvement of skin, lung, liver, GI tract, muscles, and eyes. Treatments for chronic GVHD included corticosteroids, extracorporeal photopheresis, abatacept, hydroxychloroquine, sirolimus, rituximab, ruxolitinib, belumosudil, interleukin‐2, and methotrexate (Figure 1). Axatilimab had not yet been approved by the U.S. Food and Drug Administration for chronic GVHD at the time of his presentation.

**FIGURE 2:**
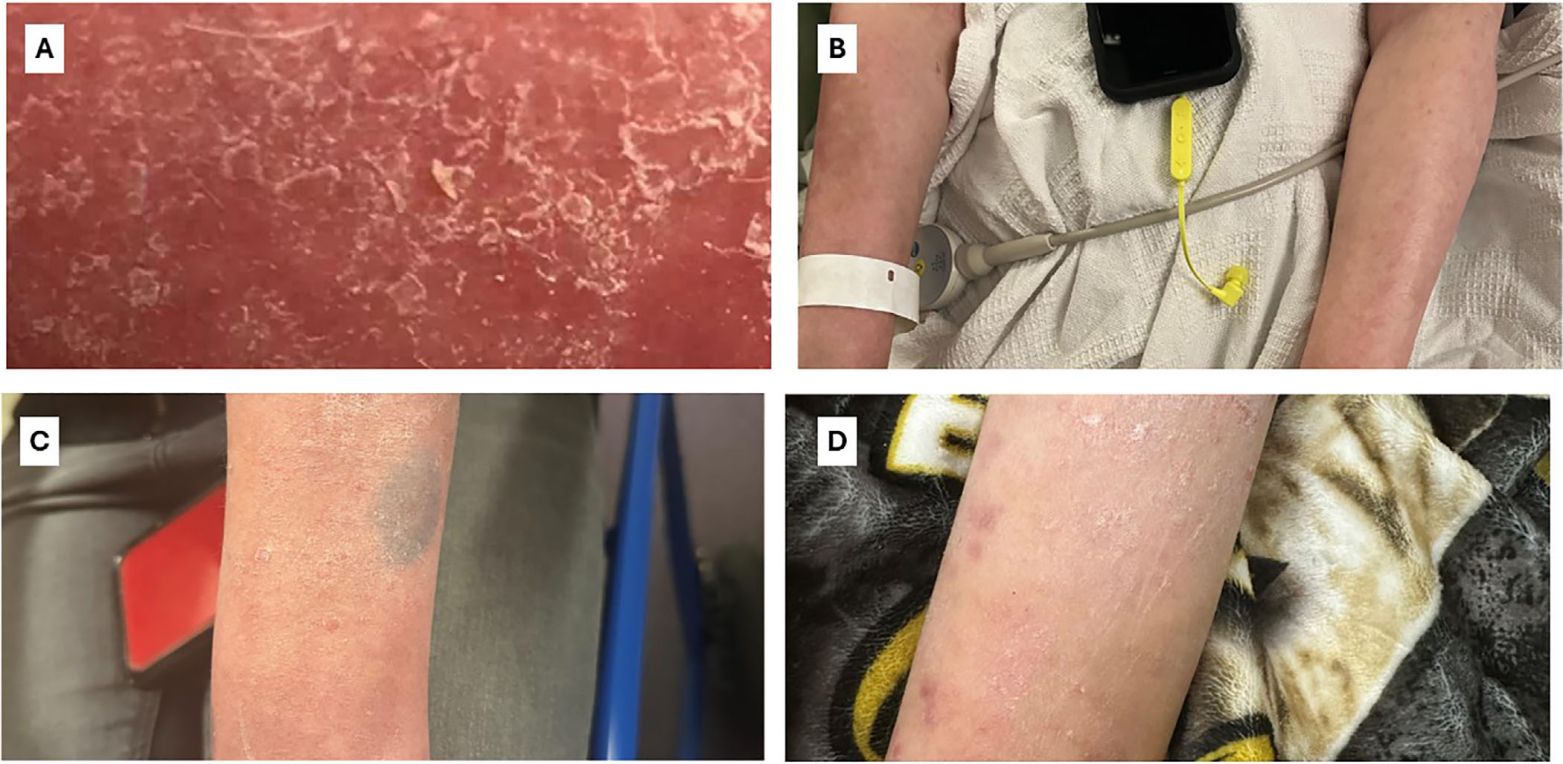
Evolution of cutaneous GVHD response to CD162 targeting. (A) Baseline skin with severe erythema, edema, weeping, and sloughing. (B) Improvement in erythema 3 weeks after the first dose of ALTB‐168, but edema remains. (C) Resolution of edema but mild erythema persists prior to ALTB‐268. (D) Complete resolution of erythematous inflammatory dermatitis after ALTB‐268. Only a slight dry scale remains.

His course was marked by profound morbidity. Complications included adrenal insufficiency, severe hypogammaglobulinemia with multiple recurrent infections, malnutrition, venous thromboembolism, avascular necrosis (AVN) of hips and knees due to steroids, osteoporosis, sarcopenia, and marked debility. Cutaneous GVHD was the dominant and most disabling manifestation. He developed recurrent, abrupt inflammatory flares characterized by diffuse erythema, scaling, pain, and marked skin fragility (Figure 2A). Severe episodes required opioid analgesia, intensive skin care, and substantial caregiver support, with major impairment in mobility and activities of daily living.

Between 2021 and 2024, his disease remained nearly continuous, punctuated by severe flares frequently triggered by bacterial or fungal infections. These episodes repeatedly required prolonged inpatient treatment. Hospitalization duration for his last flare prior to compassionate use anti‐CD162 therapy exceeded 1 year, underscoring the exceptional severity of his illness and the sustained burden on the patient and caregivers. Skin biopsies obtained during active flares demonstrated inflammatory changes consistent with chronic GVHD, including lichenoid and psoriasiform features, supporting a highly inflammatory and treatment‐refractory phenotype.

## 3 Experimental Intervention

Given persistent severe disease activity despite ongoing therapy with multiagent therapy with corticosteroids, belumosudil, abatacept, and a recent course of oral methotrexate, ALTB‐168 was obtained under an institutional review board‐approved compassionate‐use protocol and initiated in May 2024 as an intravenous regimen. After an initial intravenous dose of 6 mg/kg, weekly 4 mg/kg doses were given, then transitioned to 4 mg/kg every other week, and subsequently to 4 mg/kg monthly for maintenance. He received a total of 10 doses of ALTB‐168 until the global experimental drug supply was exhausted. Given the ongoing clinical benefit with very good partial response, followed by skin GVHD flare when ALTB‐168 was no longer available, we sought to provide him with subsequent 500 mg fixed doses of ALTB‐268, starting in July 2025. The treatment plan was 500 mg SQ weekly dosing for 4 doses to regain disease control, then every 2‐week 500 mg SQ dosing for 8 doses, and then 500 mg SQ monthly for 8 doses. He has received 16 doses of SQ ALTB‐268 to date.

## 4 Outcomes

**FIGURE 3:**
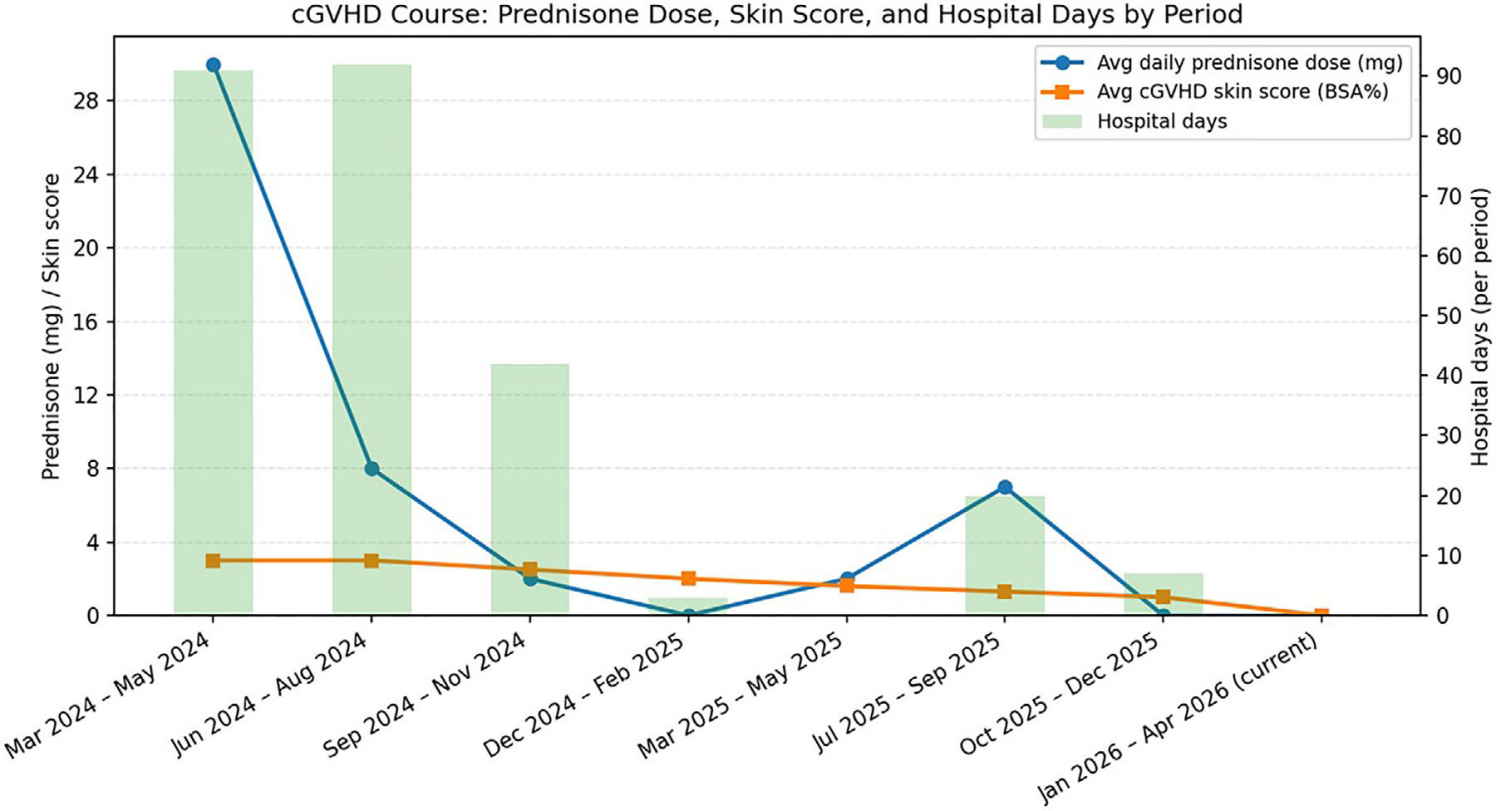
Symptom burden and steroid dose lowering in response to CD162 targeting.

After ALTB‐168 initiation, the frequency and severity of inflammatory cutaneous flares markedly declined. He showed improvement in erythema 3 weeks after the initial infusion (Figure 2B). Over the subsequent months, he was able to be dismissed from the hospital, his anasarca also improved (Figure 2C). The patient's anasarca was considered multifactorial in etiology, attributed primarily to severe hypoalbuminemia from a combination of chronic inflammation, malnutrition, and gastrointestinal protein loss in the setting of active GVHD, compounded by capillary leak from widespread inflammatory cutaneous disease and prolonged immobility. Active dermatitis resolved (Figure 2D), with only some residual skin dryness, skin thinning, and resolving purpura at most recent follow up. He was tapered off all immunosuppression, except for maintaining belumosudil as a single systemic agent, oral beclomethasone for nausea, and adrenal replacement‐dose corticosteroids. Importantly, the benefit extended beyond skin response. Mobility and daily functioning improved substantially, and he underwent bilateral (sequential) total hip arthroplasties for AVN without major infectious complications. No unexpected adverse events were attributed to ALTB‐168 or ALTB‐268. His quality of life and healthcare burden dramatically improved (Figure 3). Regarding non‐cutaneous organ involvement, his disease remained stable to improved throughout the treatment period, without clinically significant flare. Hepatic and gastrointestinal GVHD had been largely quiescent prior to initiation of anti‐CD162 therapy, managed with the concurrent immunosuppressive regimen.

## 5 Discussion

This case highlights the extreme morbidity of refractory cutaneous chronic GVHD. In this patient, skin disease was a life‐threatening inflammatory process associated with severe pain, barrier compromise, recurrent infection, prolonged deconditioning, and hospitalization exceeding 1 year. The case also illustrates a central challenge in advanced GVHD: The patients with the greatest need for disease control are often those least able to tolerate additional broad immunosuppression due to extensive complications [9, 10]. The response observed here is notable as it followed years of persistent GVHD activity and failure of multiple standard therapies. Although causal inference is limited by the single‐patient design and concurrent therapies, the temporal association between anti‐CD162 exposure and durable remission is clinically compelling, particularly given the absence of prior sustained control.

Targeting CD162 offers a mechanistically distinct strategy for inflammatory chronic GVHD, with clinical benefit observed in this case. Selective modulation of activated T cells, rather than broad immunosuppression, may be especially advantageous in heavily pretreated patients with significant infectious vulnerability. In this context, the patient's remission, corticosteroid reduction, and substantial functional recovery support further evaluation of CD162‐directed therapy in refractory chronic GVHD. Preclinical data, together with the clinical observation reported here, support the development of a prospective clinical trial evaluating CD162‐directed therapy in patients with refractory GVHD. ALTB‐268 is currently under investigation in a Phase 2a clinical trial for moderately to severely active ulcerative colitis refractory to biologic therapy (NCT06109441), and indication expansion to other T cell‐mediated inflammatory diseases including GVHD is under consideration.

6

A.L. served as co‐investigator on the protocol, collected data, and edited the manuscript. R.M. collected data and edited the manuscript. M.R. analyzed data and edited the manuscript. P.M. analyzed data and edited the manuscript. I.C. provided study drug and edited the manuscript. S.R. provided study drug and edited the manuscript. S.‐Y.L. provided study drug and edited the manuscript. J.C. provided study drug and edited the manuscript. S.G.H. was the principal investigator of the protocol, collected and analyzed data, and wrote the manuscript.

## Funding

This study drug was provided by Altrubio, Inc. There was no other funding for this study.

## Ethics Statement

This treatment was conducted under an institutional review board and U.S. Food and Drug Administration approved single‐patient investigational new drug protocol.

## Consent

The patient provided written informed consent for this treatment and sharing of information.

## Conflicts of Interest

Iming Cho, Simona Reed, Shih‐Yao Lin, and Judy Chou are employed by Altrubio, Inc. The Roswell Park coauthors have no conflict of interest with this company or protocol.

## Data Availability

Reasonable requests for data can be sent to the corresponding author.
